# Genetic diversity may help evolutionary rescue in a clonal endemic plant species of Western Himalaya

**DOI:** 10.1038/s41598-021-98648-8

**Published:** 2021-10-01

**Authors:** Irshad Ahmad Sofi, Irfan Rashid, Javaid Yousuf Lone, Sandhya Tyagi, Zafar A. Reshi, Reyazul Rouf Mir

**Affiliations:** 1 Department of Botany, University of Kashmir, Srinagar, Jammu and Kashmir 190006 India; 2 Department of Plant Physiology, Indian Agricultural Research Institute, New Delhi, Delhi 110012 India; 3 Division of Genetics and Plant Breeding, Faculty of Agriculture, SKUAST-Kashmir, Wadura Campus, Sopore, Jammu and Kashmir 193201 India

**Keywords:** Biodiversity, Climate-change ecology

## Abstract

Habitat loss due to climate change may cause the extinction of the clonal species with a limited distribution range. Thus, determining the genetic diversity required for adaptability by these species in sensitive ecosystems can help infer the chances of their survival and spread in changing climate. We studied the genetic diversity and population structure of *Sambucus wightiana*—a clonal endemic plant species of the Himalayan region for understanding its possible survival chances in anticipated climate change. Eight polymorphic microsatellite markers were used to study the allelic/genetic diversity and population structure. In addition, ITS1–ITS4 Sanger sequencing was used for phylogeny and SNP detection. A total number of 73 alleles were scored for 37 genotypes at 17 loci for 8 SSRs markers. The population structural analysis using the SSR marker data led to identifying two sub-populations in our collection of 37 *S. wightiana* genotypes, with 11 genotypes having mixed ancestry. The ITS sequence data show a specific allele in higher frequency in a particular sub-population, indicating variation in different *S. wightiana* accessions at the sequence level. The genotypic data of SSR markers and trait data of 11 traits of *S. wightiana*, when analyzed together, revealed five significant Marker-Trait Associations (MTAs) through Single Marker Analysis (SMA) or regression analysis. Most of the SSR markers were found to be associated with more than one trait, indicating the usefulness of these markers for working out marker-trait associations. Moderate to high genetic diversity observed in the present study may provide insurance against climate change to *S. wightiana* and help its further spread.

## Introduction

Climate warming is affecting the biodiversity and functioning of plant communities across the globe, and in response to this changing climate, species are now shifting their geographical distributions and expanding their ranges across elevational and latitudinal gradients^1,2^. This range expansion can increase the survival chances of species that might get affected by climate change driven shrinkage of suitable habitats; however, individual species' range responses vary greatly, with some species changing their ranges and/or local abundances rapidly while others do so slowly or not at all^3,4^. A growing body of literature seeks to explain this variation in range shifts using species’ ecological and life-history traits, with expectations that these shifts are determined by the capacity of species to disperse, establish new populations, and proliferate in the new environments^5,6^. In this race for survival, clonal plants appear to be at a particular disadvantage due to their limited mobility and limited capacity for adaptation^7^. Although, clonal plants have persisted for thousands, or even millions of years during past environmental changes^8^, the future climate change is predicted to advance much faster than during post-glacial times^9^. If a species can neither adapt to the modified environmental conditions nor migrate fast enough, then population extirpation or in the worst-case extinction of entire species is expected^10,11^. This extinction can be avoided if the populations undergo evolutionary adaptation^12–14^; however, the evolutionary potential of a population in turn depends on the pre-existing genetic variation within the species, and a high level of standing variation may allow a faster response to environmental changes^15,16^. Thus, empirical studies that estimate genetic diversity in clonal species with limited distribution ranges are important for understanding their possible survival chances in anticipated climate change.

Although the advent of new molecular tools had a major impact on the study of clonality in plant species, the results from genotyping have quite often been interpreted without consideration of the restrictions of the genetic markers used^17^, probably resulting in biased estimation of the extent of clonality. Moreover, the low resolution of allozyme markers often leads to an overestimation of the extent of clonality^18^. On the other hand, due to failure to account for small differences between multi-locus genotypes because of PCR and scoring errors or somatic mutations, the use of high-resolution markers such as Amplified Fragment Length Polymorphisms (AFLPs) may lead to an under-estimation of the extent of clonality^19^. Some molecular markers, e.g., Simple Sequence Repeats (SSRs) have been the marker of choice due to their high polymorphism detection, high reproducibility, co-dominant nature, cost-effectiveness, and ease of study^20^. In addition to molecular markers, Internal Transcriber Spacers (ITS) have been frequently used for phylogenetic analysis^21^ and the study of genetic diversity. ITS ribosomal DNA length variants have been reported for several plant species^22,23^. In the present study, eight SSR markers, initially designed for *Sambucus nigra*, and ITS1–ITS4 region Sanger sequencing, were used to quantify the genetic diversity of *Sambucus wightiana* (an endemic clonal plant species) to predict its survival chances and potential spread during anticipated climate change. The species is commonly found in forest gaps making way into the canopy, threatening the suitable habitat of many important medicinal plants, decreasing understory plant diversity, and potentially hindering the natural regeneration of conifer saplings.

## Results

### Allelic and gene diversity

SSR markers used during the present study successfully amplified expected genomic regions in a set of 37 *S. wightiana* accessions/genotypes. Multiple loci were amplified by most of the SSR markers as evident by the fact that these 8 SSR makers amplified 17 loci ranging from 1 locus to 3 loci with an average of 2.12 loci per SSR marker. While counting the number of alleles per marker, it was revealed that a total of 73 alleles were amplified by all the 8 SSR markers using 37 *S. wightiana* genotypes. The number of alleles varied from 7 for SSR marker “EMSn016” and “EMSn003” to 1 for SSR marker “EMSn023” and “EMSn025” with an average of 9.12 alleles/marker. The average number of alleles detected per loci was 4.29 (Total alleles = 73/Total loci = 17) and the percent polymorphic loci was 70.59%. The analysis of gene diversity data revealed moderate gene diversity (*He*) noticed for all SSR marker loci, which varied from 0 (for monomorphic loci) and 0.818 with an average of 0.526 (SE = 0.086). Except for the monomorphic loci of Sn023 and Sn025 the average *He* was high with an average of 0.745. The mean values *uHe* and I were 0.534 (SE = 0.087) and 1.086 (SE = 0.179) respectively (Table 1). Further analysis of marker data based on 37 genotypes of 7 watersheds is presented in Table 2. The analysis revealed that the average number of alleles (Na) ranged from 1.412 for sub-population 6, 7 to 3.059 for sub-population 1. The private alleles were found in only 2 populations: Pop1 and Pop4 with 0.143 and 0.167 frequency with an overall mean of 0.11. The mean effective number of alleles ranged from 1.412 to 2.458, which were comparable to the average number of alleles. The gene diversity (*He*) and *uHe* values ranged from 0.206 to 0.445 and 0.275 to 0.475 respectively. Also, Shannon’s information index (I) was calculated for populations with the highest diversity found in Pop1 (I = 0.828, SE = 0.151) and lowest in Pop6 (I = 0.285, SE = 0.085).

**Table 1:** Genetic diversity estimates for *Sambucus wightiana* at seventeen loci and averaged over all sub-populations.

Primer		Primer sequences	Locus	N	Na	Ne	I	He	uHe	nP	PIC = 2 × F (1 − F)
EMSn002	F	AACACTAGAAACATAAATTCAAAGTGG	Sn002a	33	5	3.351	1.369	0.702	0.712	0	0.2571
	R	TAATTCTCATTCGCGGTTCG	Sn002b	23	5	3.861	1.458	0.741	0.757	0	0.3403
			Sn002c	37	5	3.283	1.397	0.695	0.705	0	0.2337
EMSn003	F	TCGTCTTTTCCGACTCTAAAGC	Sn003a	36	7	5.492	1.811	0.818	0.829	0	0.3133
	R	CTGGACATTTGCGATCTGG	Sn003b	35	5	3.267	1.39	0.694	0.704	0	0.2449
EMSn010	F	ACATCAAACCCTGCAACACC	Sn010a	34	5	3.959	1.473	0.747	0.759	0	0.2509
	R	TCGCTCTAATTCCAACATTGC	Sn010b	34	5	3.026	1.32	0.67	0.68	0	0.2509
EMSn016	F	GGCGCAGACCAATTATAACATAG	Sn016a	31	6	5.491	1.744	0.818	0.831	0	0.3122
	R	TCCACCATTCTTCCTTCTGC	Sn016b	27	7	4.893	1.726	0.796	0.811	2	0.3841
			Sn016c	33	6	5.161	1.705	0.806	0.819	0	0.2975
EMSn017	F	GGTATTGCTTGAACAATCATCG	Sn017	33	6	3.138	1.441	0.681	0.692	0	0.2975
	R	GCCTTTTGCCCAAACTATCC									
EMSn019	F	GGTGAAACTTGAAAATCCTAGCC	Sn019	37	6	4.43	1.63	0.774	0.785	0	0.2717
	R	GGTCCGAAATAGAACACTAAAGC									
EMSn023	F	TGGCATTTGTTTAATGATCACG	Sn023a	28	1	1	0	0	0	0	0.0689
	R	TTTAAAGAAGGGGTGAACACG	Sn023b	28	1	1	0	0	0	0	0.0689
EMSn025	F	AATGCATCGCAAGAAAAAGG	Sn025a	31	1	1	0	0	0	0	0.0624
	R	GGTAAGATAAATGATACAATGTTTTGG	Sn025b	31	1	1	0	0	0	0	0.0624
			Sn025c	30	1	1	0	0	0	0	0.0644
			Mean	31.82	4.294	3.197	1.086	0.526	0.534	0.11	0.222
			SE	0.92	0.554	0.402	0.179	0.086	0.087	0	

N; Sample size, Na; No. of Alleles, Ne; No. of Effective Alleles, I; Information Index, He; Expected Heterozygosity, uHe; Unbiased Expected Heterozygosity, nP; Number of Private Alleles and PIC; Polymorphic Information Content.

**Table 2:** Mean allelic patterns across populations of *Sambucus wightiana*.

Population	Na (SE)	Na Freq. > = 5% (SE)	Ne (SE)	I (SE)	Np (SE)	No. LComm alleles (< = 50%) (SE)	He (SE)	uHe (SE)
Pop1	3.059 (0.406)	3.059 (0.406)	2.458 (0.316)	0.828 (0.151)	0.059 (0.059)	1.529 (0.298)	0.445 (0.078)	0.475 (0.083)
Pop2	2.294 (0.306)	2.294 (0.306)	2.140 (0.292)	0.647 (0.136)	–	1.059 (0.218)	0.384 (0.075)	0.443 (0.086)
Pop3	2.235 (0.291)	2.235 (0.291)	1.786 (0.192)	0.554 (0.124)	–	0.882 (0.270)	0.324 (0.070)	0.345 (0.075)
Pop4	2.529 (0.344)	2.529 (0.344)	2.136 (0.273)	0.687 (0.136)	0.059 (0.059)	1.000 (0.257)	0.397 (0.072)	0.429 (0.078)
Pop5	1.706 (0.187)	1.706 (0.187)	1.647 (0.183)	0.422 (0.107)	–	0.471 (0.194)	0.278 (0.068)	0.337 (0.083)
Pop6	1.412 (0.123)	1.412 (0.123)	1.412 (0.123)	0.285 (0.085)	–	0.471 (0.174)	0.206 (0.062)	0.275 (0.082)
Pop7	1.412 (0.173)	1.412 (0.173)	1.412 (0.173)	0.367 (0.086)	–	0.471 (0.151)	0.265 (0.062)	0.353 (0.083)

Na = No. of Different Alleles.

Na (Freq >  = 5%) = No. of Different Alleles with a Frequency >  = 5%

Ne = No. of Effective Alleles = 1/(Sum pi^2).

I = Shannon's Information Index =  − 1* Sum (pi * Ln (pi)).

No. Private Alleles = No. of Alleles Unique to a Single Population.

No. LComm Alleles (< = 25%) = No. of Locally Common Alleles (Freq. >  = 5%) Found in 25% or Fewer Populations.

No. LComm Alleles (< = 50%) = No. of Locally Common Alleles (Freq. >  = 5%) Found in 50% or Fewer Populations.

He = Expected Heterozygosity = 1 − Sum pi^2.

uHe = Unbiased Expected Heterozygosity = (2 N/(2 N − 1)) * He.

### Clustering and principal coordinate analysis (PCoA)

To understand the patterns of variations of genetic diversity by SSR data, two multivariate methods were employed; clustering and principal coordinate analysis.

### Clustering

**Figure 1:**
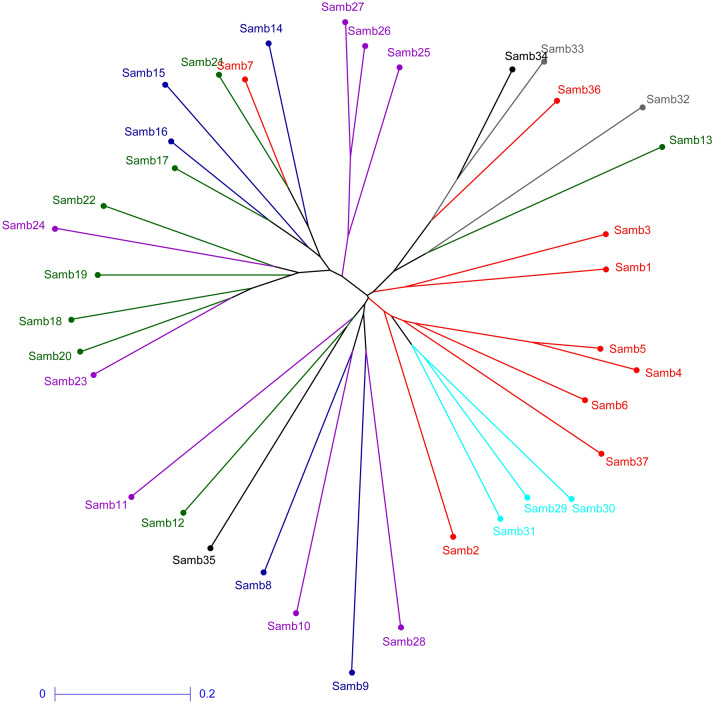
UNJ dendrogram showing clustering pattern of *S. wightiana* Samples (DARwin ver. 6.0; http://darwin.cirad.fr/darwin).

The clustering analysis of the 37 genotypes collected across the geographical spectrum of the *S. wightiana* in the Kashmir valley revealed three main clusters (Fig. 1). Cluster I contain two small sub-clusters Ia (Samb-1 and Samb-3) and sub-cluster Ib. The sub-cluster Ib is further divided into 2 more small clusters Ib1 (Samb-13 and Samb-32) and Ib2 (Samb-33, 34, and 36). Cluster II is sub-divided into two clusters, sub-cluster IIa (Samb-25, 26, and 27) and sub-cluster IIb. The sub-cluster IIb is further divided into two small clusters cluster IIb1 (Samb-7, 14, 15, 16, 17, and 21) and IIb2 (Samb-11, 18, 19, 20, 23, 24) with several smaller sub-clusters. Cluster III is divided into two sub-clusters, sub-cluster IIIa and sub-cluster IIIb. Sub-cluster IIIa is further divided into two smaller clusters cluster IIIa1 (samb-11, 12, and 35) and cluster IIIa2 (Samb-8, 9, 10, and 28). Sub-cluster IIIb is further divided into IIIb1 (Samb-2) and IIIb2 (samb-4, 5, 6, 29, 30, 31, and 37). The clustering does not show any rigid correspondence between genotypes and their geographic location.

### Principal co-ordinate analysis (PCoA)

**Figure 2:**
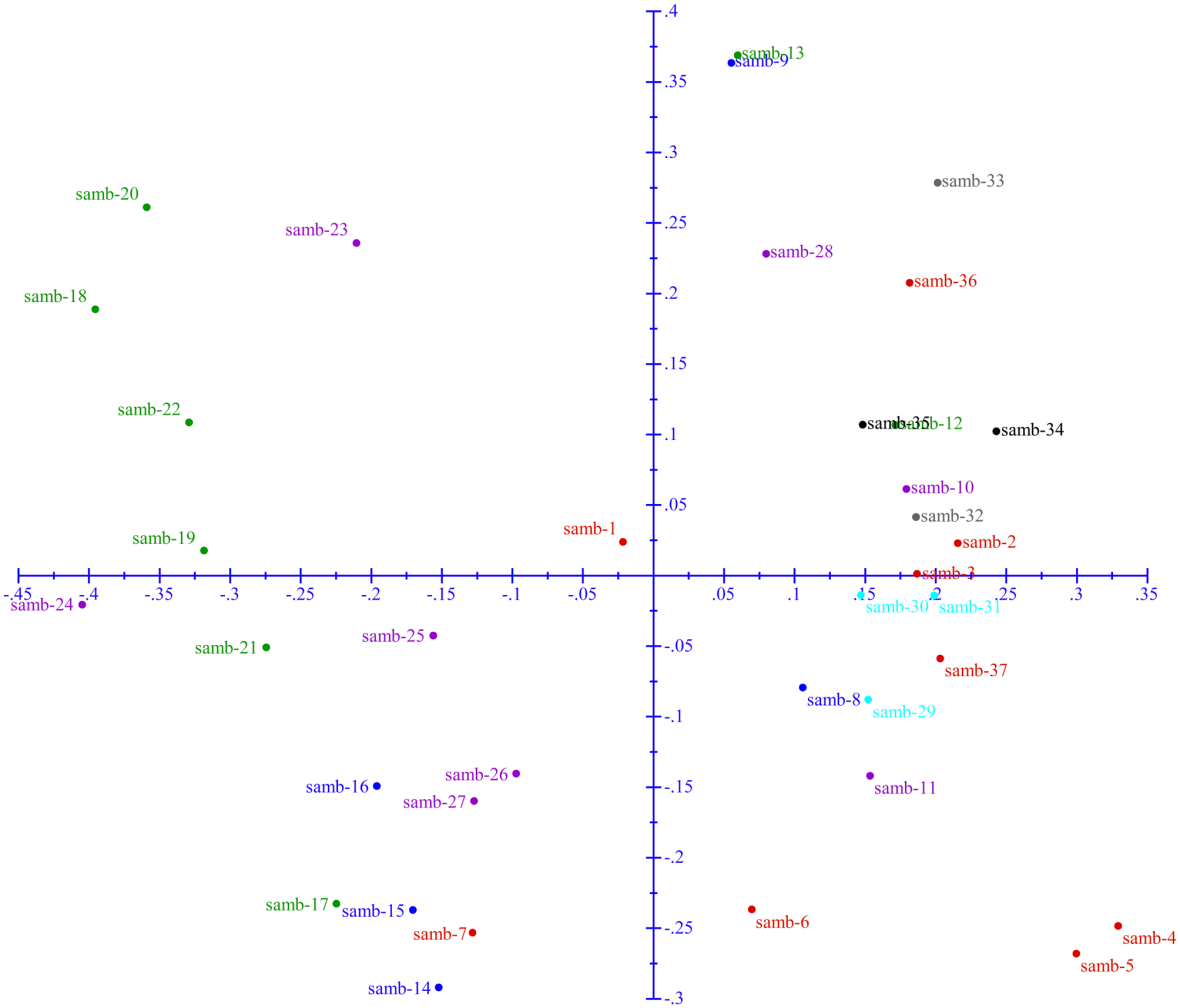
Results of the principal coordinates analysis of 17 microsatellite loci in *S. wightiana* (DARwin ver. 6.0; http://darwin.cirad.fr/darwin).

Principal Coordinate Analysis (PCoA) was carried by using the data matrix/scores (1-presence and 0-absence) of all 8 SSR markers that led to the uniform spread of all 37 *S. wightiana* genotypes into all 4 coordinates (Fig. 2). Principal Coordinates Analysis (PCoA) could not identify significant isolation in populations as all genotypes were widely distributed in all the quadrants (I and IV) of factorial analysis. PCoA could not identify any significant isolation and rigid assemblies of genetic proximity to spatial distribution.

### The analysis of molecular variance (AMOVA)

AMOVA was carried to analyze the distribution of genetic variation among sub-population from 7 different watersheds representing the different populations and among and within individuals/accessions/genotypes. The AMOVA revealed that the majority of the genetic variation is partitioned among individuals (i.e., within site/population) i.e., 90% and only 10% variation is present among populations (Table 3). Based on AMOVA the genetic differentiation values for global Fst, Fst max, and F’st were 0.104, 0.403, and 0.258 respectively indicating a moderate level of genetic differentiation among populations. Pairwise Fst genetic distances between each pair of population/subpopulations were also estimated. The values for pairwise Fst between populations/ sub-populations 1–4 and 6–7 were high (0.125 and 0.417 respectively) with an average of 0.285 (Table 4). Pairwise Nei’s Genetic Distance, Nei’s Genetic identity, and corresponding unbiased Nei’s Genetic Distance and Nei’s Genetic identity values (Table 5) were in agreement with pairwise Fst calculations reflecting a moderate genetic differentiation among populations. Chi-Square Tests for Hardy–Weinberg Equilibrium revealed the probability at all loci except for 2 monomorphic loci; Sn023 and Sn025 were statistically significant (Table 6) confirming the deviation from the HWE and nonrandom mating of *S. wightiana* with subtle signs of inbreeding.

**Table 3:** Summary of analysis of molecular variance (AMOVA).

Source	df	SS	MS	Est. Var	Variation explained (%)
Among Pops	6	96.302	16.050	0.588	10.5
Among Indiv	30	303.705	10.123	5.059	89.4
Within Indiv	37	0.220	0.006	0.006	0.1
Total	73	400.226		5.653	100

**Table 4:** Pairwise population Fst values.

	Pop1	Pop2	Pop3	Pop4	Pop5	Pop6	Pop7
Pop1	0.000						
Pop2	0.161	0.000					
Pop3	0.182	0.224	0.000				
Pop4	**0.125**	0.198	0.146	0.000			
Pop5	0.212	0.295	0.311	0.271	0.000		
Pop6	0.299	0.354	0.381	0.387	0.317	0.000	
Pop7	0.269	0.382	0.405	0.306	0.348	**0.417**	0.000

Maximum and minimum Fst values are given in bold.

**Table 5:** Genetic Diversity Indices analyzed in *S. wightiana* populations; Pairwise Population Matrix of (a) Nei’s Genetic Distance, (b) Nei’s Unbiased Genetic Distance, (c) Nei’s Genetic Identity and (d) Nei’s Unbiased Genetic Identity.

	Pop1	Pop2	Pop3	Pop4	Pop5	Pop6	Pop7		Pop1	Pop2	Pop3	Pop4	Pop5	Pop6	Pop7
**a**								**b**							
Pop1	0							Pop1	0						
Pop2	0.316	0						Pop2	0.238	0					
Pop3	0.307	0.357	0					Pop3	0.264	0.29	0				
Pop4	0.248	0.372	**0.194**	0				Pop4	0.194	0.294	**0.15**	0			
Pop5	0.356	0.49	0.517	0.444	0			Pop5	0.286	0.396	0.458	0.374	0		
Pop6	0.48	0.555	0.613	**0.682**	0.358	0		Pop6	0.408	0.46	0.552	**0.61**	0.27	0	
Pop7	0.343	0.636	0.616	0.416	0.361	0.496	0	Pop7	0.339	0.609	0.623	0.412	0.341	0.474	0
**c**								**d**							
Pop1	1							Pop1	1						
Pop2	0.729	1						Pop2	0.788	1					
Pop3	0.735	0.7	1					Pop3	0.768	0.748	1				
Pop4	0.78	0.689	**0.824**	1				Pop4	0.824	0.745	**0.86**	1			
Pop5	0.7	0.613	0.596	0.642	1			Pop5	0.751	0.673	0.632	0.688	1		
Pop6	0.619	0.574	0.542	**0.505**	0.699	1		Pop6	0.665	0.632	0.576	0.543	0.763	1	
Pop7	0.71	0.529	0.54	0.659	0.697	0.609	1	Pop7	0.712	0.544	**0.537**	0.662	0.711	0.622	1

Maximum and minimum values for different diversity indices; a, b, c, and d are highlighted in bold.

**Table 6:** Summary of chi-square tests for Hardy–Weinberg equilibrium.

Pop	Locus	DF	ChiSq	Prob	Signif
Pop1	Sn016a	15	155.000	0.000	***
Pop1	Sn016b	21	162.000	0.000	***
Pop1	Sn016c	15	165.000	0.000	***
Pop1	Sn010a	10	136.000	0.000	***
Pop1	Sn010b	10	136.000	0.000	***
Pop1	Sn017	15	165.000	0.000	***
Pop1	Sn019	15	185.000	0.000	***
Pop1	Sn023a	Monomorphic			
Pop1	Sn023b	Monomorphic			
Pop1	Sn025a	Monomorphic			
Pop1	Sn025b	Monomorphic			
Pop1	Sn025c	Monomorphic			
Pop1	Sn02a	10	132.000	0.000	***
Pop1	Sn02b	10	92.000	0.000	***
Pop1	Sn02c	10	148.000	0.000	***
Pop1	Sn03a	21	216.000	0.000	***
Pop1	Sn03b	10	140.000	0.000	***

Key: ns = not significant, *P < 0.05, **P < 0.01, ***P < 0.001.

### ITS region sequence analysis

The Internal Transcribed Spacer (ITS) region that lies between nuclear small rDNA and nuclear large rDNA is considered most important for phylogenetic inference at the generic and intrageneric levels in plants (Alvarez and Wendel 2003). The ITS sequence data has been used in ~ 66% of studies to analyze the phylogeny and 34% of all published phylogenetic hypotheses have been based exclusively on ITS sequences^23,24^. Therefore, efforts have been made during the present study to sequence the ITS region of 37 *S. wightiana* accessions/genotypes collected from the North-Western Himalayas of Jammu and Kashmir. The sequence data generated during the present study was also compared with ITS region sequencing data of 18 other *Sambucus* species including *S. wightiana*. The analysis of sequencing data led to the identification of 10 SNPs (SNP density: 1 SNP/ 57.2 bp) in the 645 bp sequence of 37 *S. wightiana* genotypes. Among the 10 SNPs, five SNPs were found very promising (Table 7), and the frequency of these SNPs was either 20% or > 20%. In addition, one insertion has also been noticed with a frequency of 34%. A large number of SNPs were also noticed while comparing the *S. wightiana* genotypes with the ITS sequences of 18 other *Sambucus* species. One large insertion of ~ 25 bp was identified in *S. wightiana* or deletion of 25 bp in other 18 *Sambucus* species. Sequence variation between different genotypes has been identified at ITS sequence in several earlier studies in different plants including *Cinnamomum*^25^, Jujube^26^, Common bean^21,24^, Coneflowers and Relatives^27^, and *Sambucus* and *Adoxa*^28^. The important SNPs identified during the present study could be converted into user-friendly PCR-based markers for species discrimination, genetic characterization, and phylogeny analysis.

**Table 7:** List of SNPs detected in the ITS sequences of *S. wightiana*.

Genotype	SNP/insertion position										
	44	108	157	195	451	453	454	457	461	462	Insertion at 447
Samb-32	G	A	C	T	G	C	T	G	A	T	–
Samb-28	G	G	C	T	G	C	T	G	A	T	–
Samb-30	T	A	C	T	G	A	G	G	A	T	–
Samb-26	G	G	C	T	G	A	G	T	A	T	–
Samb-14	G	A	C	T	G	A	G	G	A	T	–
Samb-5	G	A	C	T	G	A	G	G	A	T	–
Samb-8	G	A	C	T	G	A	G	G	A	T	–
Samb-3	T	A	C	T	G	A	G	G	A	T	–
Samb-9	T	A	C	T	G	A	G	G	A	T	–
Samb-16	G	A	C	T	G	C	G	G	A	T	–
Samb-27	G	A	C	T	G	C	G	G	A	T	–
Samb-35	G	G	C	T	G	C	G	G	A	T	–
Samb-15	G	A	C	T	G	C	G	G	A	T	–
Samb-2	G	A	C	T	G	C	G	G	G	T	–
Samb-36	G	A	C	T	G	C	G	G	G	T	–
Samb-4	G	A	C	T	G	C	G	G	G	T	–
Samb-20	G	G	C	T	G	C	G	G	G	T	T
Samb-17	G	G	C	T	G	C	G	G	G	T	T
Samb-18	G	G	C	T	A	C	T	G	A	C	T
Samb-24	G	G	C	T	A	C	T	G	A	C	G
Samb-19	G	G	C	T	A	C	T	G	A	T	G
Samb-13	T	G	C	T	A	C	T	G	G	C	T
Samb-12	T	G	G	T	A	C	T	G	G	T	T
Samb-29	G	G	C	T	G	C	G	G	G	T	T
Samb-22	T	G	C	T	G	C	G	G	G	T	T
Samb-23	T	G	C	T	G	C	G	G	G	T	T
Samb-21	G	G	C	T	G	C	G	G	G	T	T
Samb-25	G	G	C	T	G	C	G	G	G	T	T
Samb-37	G	A	C	T	G	C	G	G	G	T	–
Samb-10	G	A	C	C	G	C	G	G	G	T	–
Samb-6	G	A	C	T	G	C	G	G	A	T	–
Samb-33	G	A	C	T	G	C	T	G	A	T	–
Samb-7	G	A	C	T	G	A	G	G	A	T	–
Samb-31	G	A	C	T	G	A	G	G	A	T	–
Samb-34	G	A	C	T	G	A	T	T	A	T	–
	28	20	34	34	30	25	26	33	21	32	23
SNP/insertion counts	7	15	1	1	5	10	9	2	14	3	10 + 2
SNP frequency	20.00	42.86	2.86	2.86	14.29	28.57	25.71	5.71	40.00	8.57	34.28
Total sequence length	630 bp										
Total SNPs/insertions	11										
SNP density	I SNP/57.2 bases										

### ITS sequences in phylogenetic analysis of *S. wightiana*

‘Fasttree’, built using online software tool “Clustal W”, with slow NNI and MLACC = 3 based on phylogenetic reconstructions using the function ‘build’ of ETE3 v.3.1.1 was comparable to MEGA 6 results. The optimal tree by MEGA 6 was constructed with the sum of branch length for ITS1 (0.48689146 for NJ and 0.52255238 for UPGMA) and ITS4 (1.61554106 for NJ and 1.80660053 for UPGMA). Both NJ and UPGMA methods show similar results, yet UPGMA showed the best fit results (Suppl S2 and S3). The results didn’t show any geographic-specific clustering among the genotypes.

The sequences of 17 other species of *Sambucus* and one *S. wightiana* sequence downloaded from gene bank (https://www.ncbi.nlm.nih.gov/genbank/) were used as outgroups genotype during the analysis. The cluster analysis led to a clear-cut separation of 17 *Sambucus* species from *S. wightiana* (Suppl S4). It is important to note that our genotypes clustered with the out-group *S. wightiana* genotype sequence downloaded from the gene bank. The results of the present study show the diverse nature of *S. wightiana* genotypes growing wild in the natural habitats of the North-Western Himalayas.

### Structure analysis

**Figure 3:**
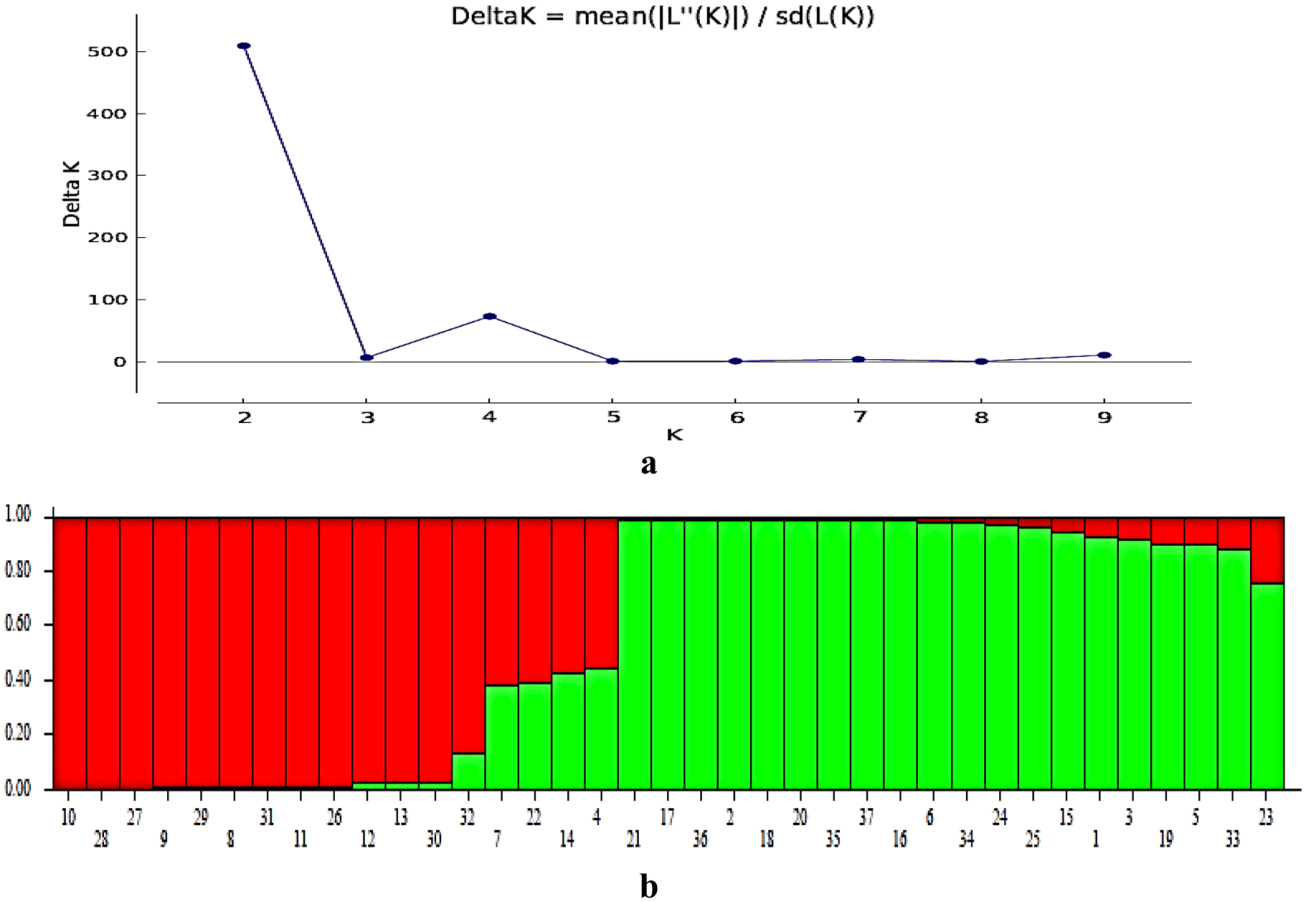
Estimation of the number of groups based on output from STRUCTURE-software (STRUCTURE 2.3.4; https://web.stanford.edu/group/pritchardlab/structuresoftware/releaseversions/v2.3.4/html/structure.html). (**a**) ΔK over K from 1 to 10 with the 8 SSR markers. (**b**) Bar plot showing grouping of 37 genotypes into two different groups.

The analysis of the population structure of 37 *S. wightiana* genotypes revealed the presence of two sub-populations (Fig. 3). Sub-population one possesses 17 genotypes, and the second sub-population possesses 20 genotypes. Among 37 genotypes, a set of 4 genotypes were found admixed *i.e.,* their affiliation probability with a particular sub-population is < 80% and these individuals tend to have mixed ancestry and tend to group with different sub-populations. The information of population structure is particularly important for working out marker-trait association and avoiding spurious associations.

### Marker-trait associations (MTAs)

The study of MTAs using the General Linear Model (GLM) and Mixed Linear Model (MLM) was analyzed in the software program TASSEL led to the identification of a total of 5 SSR markers associated with 11 traits in *S. wightiana*. Through GLM, one allele/marker each was found associated with plant height, leaf weight, and INL, two alleles/markers with RGR, stem weight, plant weight, MSL, MA, and TL, and three alleles/markers with fruit weight and rhizome weight. Marker SN-04 was found associated with 8 traits, marker SN-07 was found associated with three traits, marker SN-08 was found associated with 5 traits, marker SN-12 with 3 traits, and marker SN-06 with only one trait (Table 8), while as through MLM approach, only one marker (SN-12) was found associated with two different traits (stem weight and fruit weight). The marker SN-12 found associated with 3 traits through GLM and two traits through MLM is the most important marker/genomic loci for *S. wightiana*.

**Table 8:** Association of marker alleles with phenotypic traits using GLM and MLM.

S. No	Trait	Marker	Allele	F ratio	*P* value	R^2^ (%)
**GLM**						
1	Height	SN_4	SN_4.2	8.098	0.0015	29.1360
2	RGR	SN_7	SN_7.3	6.414	0.0047	25.5010
		SN_8	SN_8.2	6.524	0.0043	25.8085
3	Rhizome weight	SN_4	SN_4.2	7.583	0.0021	25.8065
		SN_8	SN_8.3	5.868	0.0069	21.5713
		SN_12	SN_12.6	7.710	0.0091	15.2519
4	Stem weight	SN_4	SN_4.2	8.767	0.0010	29.2205
		SN_7	SN_7.3	7.017	0.0031	25.2056
			SN_7.4	6.536	0.0043	23.9905
			SN_7.5	7.074	0.0029	25.3467
5	Leaf weight	SN_4	SN_4.2	5.729	0.0076	20.8308
6	Fruit weight	SN_4	SN_4.2	6.471	0.0045	22.2525
		SN_8	SN_8.3	6.353	0.0049	21.9648
		SN_12	SN_12.6	10.185	0.0032	18.2416
7	Plant weight	SN_7	SN_7.4	5.872	0.0069	21.5924
		SN_7	SN_7.5	5.385	0.0098	20.2637
8	MSL	SN_4	SN_4.2	8.550	0.0011	28.4979
		SN_8	SN_8.3	5.530	0.0088	21.0799
9	MA	SN_4	SN_4.2	5.388	0.0098	20.5485
		SN_12	SN_12.6	9.005	0.0052	17.4950
10	TL	SN_4	SN_4.2	10.244	0.0004	31.7693
		SN_8	SN_8.3	5.708	0.0078	21.4884
11	INL	SN_6	SN_6.1	6.916	0.0033	27.4183
**MLM**						
1	Stem weight	SN_12	SN_12.6	9.198	0.0046	24.5906
2	Fruit weight	SN_12	SN_12.6	8.848	0.0054	23.1308

With *P* value =  < 0.01.

## Discussion

Type of reproduction has an important effect on the maintenance of particular populations and species persistence in time and space. This trait significantly influences the ecological and genetic structure of populations and in consequence the evolution of species^29^. The prevailing opinion is that clonality reduces genetic diversity^30^. Many studies document lower levels of genetic variation in clonal species preceding repeated genetic bottlenecks leading to a prolonged lag phase^31,32^ and in populations of such species extinction is prevented with more intense vegetative reproduction with extensive human-mediated propagule dispersal^33–36^. In contrast to this opinion, comparable, or even higher genetic variation for 21 clonal plant species has been documented than non-clonal ones^37^. The results of many other studies also confirm this assertion, concerning the high level of genetic variation and genotypic diversity of species with clonal reproduction^35,38,39^. The most plausible explanation for high levels of genetic variation in populations of clonal plants is the presence of gene flow via long-distance dispersal and multiple introductions reducing the ecological and environmental constraints on genetic diversity whereas enhancing the potential of sexual reproduction, even if it is periodic and meagre^40–43^. Thus clonality helps in the maintenance and expansion of existing populations while the occurrence of sexual reproduction helps in the recruitment of new individuals/ populations however maintaining the genetic diversity^44^. Clonal plants are more successful in heterogeneous environments benefiting from clonal integration, which may enhance sexual fitness^45^. However, clonality does not substitute sexual mode of reproduction and genetic rather than ecological factors prove crucial for long term success^46–48^.

The well-known genetic paradox of how spreading species maintain genetic diversity despite going through founder effects and genetic bottlenecks during the range expansion^49^. However, some studies have shown spreading plant species can maintain a remarkable level of genetic diversity across distances, in such cases the gene flow via long-range seed dispersal or pollen might be more important than commonly suggested^50,51^. This may be also associated with the multiple introductions leading to diverse genetic admixtures^52–56^. Local adaptation to environmental conditions is also considered one among the other dependent factors providing an advantage to spreading species^57–62^. Genetic variation plays important role in the evolution-related traits for local adaptation^63^. Also, evolutionary modifications to reproductive systems provide the spreading capability for widespread species^64^.

The non-availability of any data on genetic diversity and the aggressive clonal nature led to the perception of low diversity in *S. wightiana*. Against the anticipated low genetic diversity in clonal plants, *S. wightiana* showed a remarkable genetic diversity which is consistent with some other studies^65,66^. The number of alleles per marker is a good indicator of genetic variability^67^. The detection of 73 alleles in 37 *S. wightiana* genotypes by 8 SSR primers with 70.59 percent polymorphic loci indicates moderate to high levels of genetic diversity. The high value of average gene diversity for all polymorphic loci (*He* = 0.74) also points to a good amount of overall genetic variability. Also, the variable ITS regions are used as a standard to measure genetic variability for a long time. The identification of several SNPs among different *S. wightiana* genotypes with SNP density of 1 SNP/57.2 bp also indicates high diversity in *S. wightiana* at the sequence level. The present study revealed a good magnitude of genetic diversity and a moderate differentiation with no geographical pattern among populations^68^.

Our study does not find any geographically distinct clustering of *S. wightiana* individuals from both clusterings, PCoA by SSR scores and phylogeny by ITS markers^69^. This can be attributed to the mixing of the genotypes across the regions via gene flow between populations^70^. AMOVA showed that most of the variation is due to individuals/ genotypes only i.e., 90 percent and only 10 percent of the variation between geographically distant populations. These results suggest the recruitment of new genotypes into the populations with equivalent genetic differentiation as of non-clonal species. Yet clonality remains a vital strategy, shown by the deviance from Hardy–Weinberg equilibrium for the studied microsatellite loci^71^. Clonality associated with perineal growth can moderate the negative effects of stochastic population events like genetic drifts, etc.^72^. This magnitude of polymorphism at the studied microsatellite loci and the resultant genetic diversity may point to the spreading success of this clonal species. The potential for both sexual and clonal reproduction of a species provides the greater ability for landscape spread^47^. Significant pairwise Fst values (0.125–0.417) were observed which agree with the Nei’s genetic distance and identity parameters, indicating the substantial differentiation between the populations^73^.

The structure analysis using SSR marker data during the present study revealed different sub-populations (two sub-populations) in our collection of 37 *S. wightiana* genotypes. The presence of two sub-populations indicated differences in allele frequency and population differentiations in different sub-populations, indicating a good diversity available in *S. wightiana* genotypes in the Western Himalayas. The analysis of genotypic data in combination with trait data of the traits led to the identification of genomic loci/genomic regions that are associated with these traits. Using different models (GLM and MLM) 5 markers/loci were found associated with 11 traits, with some markers controlling more than one trait (pleiotropy). The markers controlling more than one trait are considered the most important markers/genomic loci and may be due to pleiotropy or linkage of multiple genes. Identification of more genomic loci through GLM than MLM is attributed to more stringent criteria and incorporation of kin-ship matrix into the analysis in MLM, in addition to genotypic data, trait data, and population structure matrix. In GLM association analysis, only genotypic data, trait data, and population structural matrix are used to work out MTAs. The genes/genomic regions/loci identified will prove useful in future *Sambucus* breeding programs and provided insights into the genetics of these traits and the kind of gene action in *Sambucus*.

## Conclusion

Clonal endemic plant species like *S. wightiana* can acquire a moderate amount of genetic diversity, creating a wider genetic base that can withstand the stress environments like the conditions created by changing climate of the North-Western Himalaya. In this study, no particular genotype was found dominant across the regions. Lack of correlation between genetic diversity and geographic distances in *S. wightiana* could be associated with successful sexual reproduction between genets followed by long-range seed dispersion by human or animal agents, and subsequent recruitment in isolated *S. wightiana* populations enabling the gene flow between populations and forming diverse genetic admixtures^74^. The present study also found the successful transferability of these microsatellite markers, which could be used for further studies at a finer scale.

## Methods

### Plant species

*Sambucus wightiana* Wall. Ex Wight & Arn. is a clonal sub-shrub, mostly found in the forests of Kashmir valley within an altitudinal range of 1700–3300 m.a.s.l. Commonly called Kashmir elder, the species is a rhizomatous geophyte growing up to 2 m in height with white or yellowish-white flowers. The umbels consist of orange or red individual berries that reach full ripeness in late summer. The species is native to the North-Western Himalaya and the primary range spans from Indian administered Kashmir to Chitral in Pakistan^75^. Recently the species has been reported from other parts of the Indian Himalayas like Uttarakhand, Himachal Pradesh, and Sikkim^76^, pointing towards its spread due to adaptive evolution in response to climate change^61^. *Sambucus wightiana* is closely allied to *S. adnata* Wall., from which it differs in its glabrous or almost glabrous inflorescence.

### Study area

The present study was carried out in the Kashmir Himalaya (32° 20′ to 34° 54′ N and 73° 55′ to 75° 35′ E, total area of 15,948 km^2^), that represents a unique biogeographical province in the Indian Himalayan region^77^. The altitude of the region ranges from 1600 m.a.s.l. at Srinagar to 5420 m.a.s.l. at Kolahoi peak. Within this altitudinal range, Kashmir elder is found from 1700 to 3300 m.a.s.l. in the forests of Kashmir valley. The climate of the region is predominantly of a continental temperate type with wet and cold winters and relatively dry and hot summers. The temperature ranges from an average daily maximum of 31 °C and minimum of 15 °C during summer to an average daily maximum of 4 °C and minimum of − 4 °C during winter. The region has been reported to be a hotspot for climate change due to its complex topography, enormous glacial and water resources, and quick responding watersheds with intense seasonal and climatic variability over a small spatial scale^78^.

**Figure 4:**
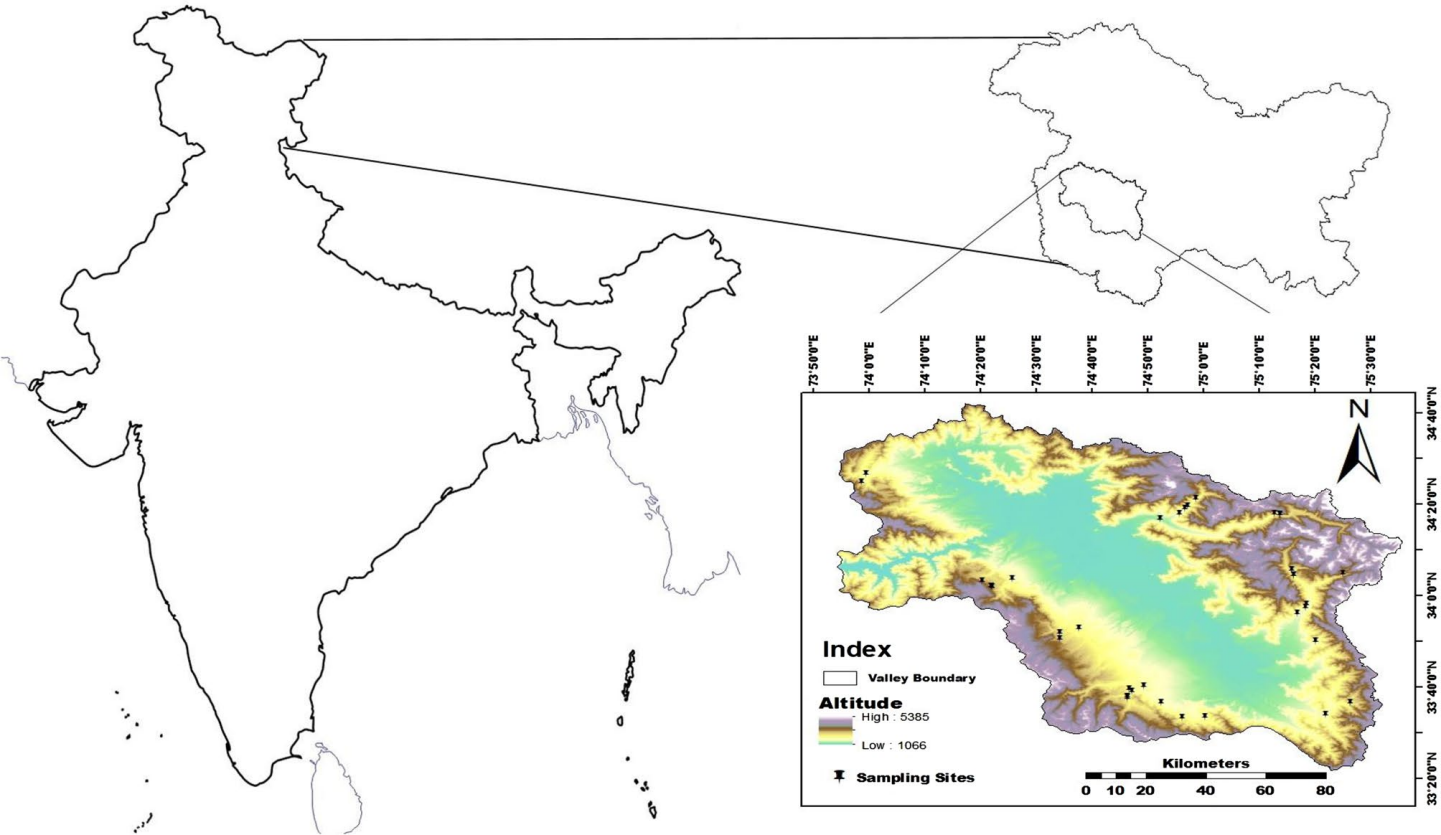
Study area showing sampling sites marked as black dots on the map inset (ArcGIS 10.2; http://resources.arcgis.com/en/help/main/10.2/index.html).

For the present study, we sampled 37 *S*. *wightiana* individuals (Table 9) from seven watersheds (Vishaw, Ferozpur, Lidder, Sindh, Doodhganga, Pohru, and Brinji) across Kashmir Himalaya, aiming to cover a wide geographic area and range of elevations (Fig. 4). The samples from each watershed were clubbed as populations for further analysis.

**Table 9:** Description of the sampling sites.

Sub-populations^a^	Sampling site	Sample code	Latitude	Longitude	Altitude (m.a.s.l.)
Vishav (Pop1)	Sedow Forest	Samb-1	33° 40.226′	74° 49.281′	2279
	Sagum Aharbal	Samb-2	33° 37.537′	74° 46.369′	2365
	Aharbal	Samb-3	33° 39.099′	74° 47.188′	2258
	Nandimarg	Samb-4	33° 33.354′	74° 56.186′	2280
	Awil	Samb-5	33° 36.580′	74° 52.471′	2066
	Dather	Samb-6	33° 33.425′	75° 00.341′	2070
	Sedow	Samb-7	33° 39.574′	74° 46.729′	2390
	Sagum Aharbal	Samb-36	33° 37.900′	74° 46.354′	2344
	Sagum Aharbal	Samb-37	33° 37.900′	74° 46.354′	2341
Ferozpur (Pop2)	Khilanmarg	Samb-8	34° 1.9572′	74° 22.0194′	3076
	Khilanmarg	Samb-9	34° 1.9572′	74° 22.0194′	3076
	Khilanmarg	Samb-14	34° 1.8706′	74° 22.080′	3042
	Gulmarg	Samb-15	34° 3.1098′	74° 20.3683′	2704
	Tangmarg	Samb-16	34° 3.5978′	74° 25.6727′	2150
Lidder (Pop3)	Mandlan	Samb-12	34° 04.545′	75° 16.147′	2351
	Mandlan	Samb-13	34° 04.545′	75° 16.147′	2351
	Chandanwari	Samb-17	34° 04.807′	75° 25.102′	2986
	Aru	Samb-18	34° 05.660′	75° 15.954′	2456
	Lidroo	Samb-19	33° 57.422′	75° 18.319′	2030
	Hapatnar	Samb-20	33° 50.031′	75° 20.194′	2070
	Langanbal	Samb-21	33° 58.053′	75° 18.506′	2022
	Laddi	Samb-22	33° 56.143′	75° 16.930′	2002
Sindh (Pop4)	Hungpark Gagangair	Samb-10	34° 17.719′	75° 13.780′	2508
	Hungpark Gagangair	Samb-11	34° 17.719′	75° 13.780′	2508
	Wangat	Samb-23	34° 19.657′	74° 57.142′	2090
	Pathkhanan	Samb-24	34° 19.044′	74° 56.662′	2048
	Burnabugh	Samb-25	34° 17.959′	74° 55.680′	2072
	Prang	Samb-26	34° 16.836′	74° 52.295′	1723
	Forest Check Point Gagangair	Samb-27	34° 17.957′	75° 12.832′	2424
	Naranag	Samb-28	34° 35.236′	74° 97.484′	2271
Doodhganga (Pop5)	RaithanTraja-Khal	Samb-29	33° 52.897′	74° 37.733′	2674
	Dangerpora Doodhpathri	Samb-30	33° 51.905′	74° 34.202′	2780
	Doodhpathri	Samb-31	33° 50.567′	74° 34.202′	2883
Pohru (Pop6)	Chokibal	Samb-32	34° 25.311′	73° 59.162′	2215
	Chokibal TP	Samb-33	34° 24.097′	73° 54.337′	2291
Brinji (Pop7)	Vailoo	Samb-34	33° 33.956′	75° 21.905′	2083
	Daksum	Samb-35	33° 36.672′	75° 26.469′	2410

^a^Based on the geographic location, all the samples from nearby locations were clubbed as populations for analysis.

### DNA extraction

Genomic DNA was extracted from the freshly collected young foliar parts of 37 *S. wightiana* accessions/genotypes by Qiagen® DNeasy™ Plant mini kit following the manufacturer’s protocol. The quality and concentration of the DNA were determined by visual comparison with the known amount of λ DNA on 0.8% agarose gel.

### Selection of SSR markers and PCR amplification

Eight polymorphic microsatellite (SSR) markers, previously developed from the *S. nigra* genome (EMSn002, 003, 010, 016, 017, 019 023, and 025) were selected, which have also been found informative in *Sambucus canadensis*^79^ (Table 1). Their sequences were downloaded from NCBI (https://www.ncbi.nlm.nih.gov/genbank/). The primers for these eight SSR markers were synthesized on contract from Sigma Aldrich, Bangalore, India. The PCR was carried out in 15 µL reaction volume with the constituents: 2 µL 20 ng DNA template, 2µL (5.0 pmol) forward and reverse primers, 0.4 µL (2.5 mM) dNTPs, 0.3 µL (1.5 U) Taq Polymerase (Sigma), 1.5 µL buffer containing MgCl_2_ with a PCR profile of; 5 min at 95 °C, 40 cycles of 95 °C for 1 min, 54 °C for 1 min 30 S of annealing temperature, 72 for 1 min and a final extension of 72 °C for 10 min in a Peqlab PCR machine.

### Selection of ITS1 and ITS4 primer sequences and their PCR

In addition to SSR markers, the ITS1-5.8S-ITS2 rDNA region was amplified using primers (ITS1: 5-GTCCACTGAACCTTATCATTTAG-3 and ITS-4: 5-TCCTCCGCTTATTGATATGC-3) synthesized on contract from Sigma Aldrich, Bangalore (for more details about primer see Choudhary et al. 2018). The PCR was done in a 25 µL reaction volume with the following constituents: 25 ng DNA template, 5.0 pmol forward and reverse primers, 2.5 mM of each dNTPs, 1X buffer, 2.0 mM MgCl_2,_ and 1.0 U of Taq DNA polymerase (Sigma) by using the following PCR profile; 5 min at 94 °C, 45 cycles of 94 °C for 30 S, 52 °C for 45 S of annealing temperature, 72 °C for 1 min and final extension at 72 °C for 5 min in a Peqlab PCR machine.

### Agarose/poly-acrylamide gel electrophoresis (PAGE)

After successful amplification, the PCR products of both ITS1–ITS4 markers and all the eight SSR markers were tested on 1.6% agarose gel to check for any amplification errors and robustness of amplification (Suppl S1a). Amplification of ITS led to a single conspicuous band of about 750 bp. The PCR product was cleaned, and Sanger sequenced by ABI 3730xl sequencer at SciGenome Genomic Services facility, Cochin, Kerala (www.scigenome.com). All SSR amplifications for 37 *S. wightiana* genotypes were subjected to PAGE analysis using PeqLab Perfect Blue Dual Gel Vertical Electrophoresis system. The gels were silver stained and digitally photographed (Suppl S1b) and the data were scored manually.

### SSR marker data analysis

For the characterization of genetic variation in the 37 *S. wightiana* genotypes and to test the transferable use of the microsatellite markers, a set of 8 SSR markers were selected. Out of the 8 SSR primers, 6 primers were polymorphic. Primers EMSn0023 and 0025 amplified a single monomorphic band. To determine the various parameters of genetic diversity, different software packages and online tools were used. The SSR marker data scored were analyzed using the software program DARwin ver. 6.0 (http://darwin.cirad.fr/darwin) ^80^, for multivariate analysis (clustering and PCoA). Both clustering and PCoA were based on Jaccard’s dissimilarity co-efficient with 1000 bootstraps. Two clustering methods (UPGMA Hierarchical clustering and Neighbor-Joining clustering) were followed. The final inferences were made on the Unweighted Neighbor-Joining clustering (UNJ), which provided the best fit to the data, and a dendrogram was constructed^81^.

Analysis of Molecular Variance (AMOVA) based on 999 permutations using FST statistics was performed with the scored SSR fragment lengths (8 markers at 17 loci) to determine the segregation of total genetic variation between, among, and within populations using GenAlEx v.6.5 (https://biology-assets.anu.edu.au/GenAlEx/Welcome.html)^82^. The parameters included the number of alleles for a given locus (N), Number of effective alleles (Ne), Shannon’s Information Index (I), Observed Heterozygosity (*Ho*), Expected Heterozygosity (*He*), Unbiased Expected Heterozygosity (*uHe*), and Fixation Index (F) along with their mean and Standard Error (SE). GenAlEx was also used to calculate the mean allelic patterns across the populations with the above-mentioned parameters.

Since our study was confined to a small number of samples, pairwise Nei’s Genetic Distance, Nei’s Genetic identity, and corresponding unbiased Nei’s Genetic Distance and Nei’s Genetic identity values were calculated across populations^83,84^. Also, Chi-Square Tests for Hardy–Weinberg Equilibrium were implemented in GenAlEx v.6.5. The Polymorphism Information Content (PIC) values of individual primers at all loci were calculated based on the formula PIC = 2 × F (1 − F)^85^.

An analysis of population structure was done using the software STRUCTURE v.2.3.4 (https://web.stanford.edu/group/pritchardlab/structure_software/release_versions/v2.3.4/html/structure.html)^86^. Population structure was analyzed by setting the number of sub-populations (k-values) from 1 to 10 and each run was repeated five times. The program was set to 300,000 burn-in iterations, followed by 600,000 Markov Chain Monte Carlo (MCMC) replications along with the admixture model. The STRUCTURE HARVESTER web version v0.6.94 (http://taylor0.biology.ucla.edu/structureHarvester/)^87^ was used to derive the appropriate number of sub-populations using a modified delta K (∆K) method^88^.

A study of Marker-Trait Associations (MTAs) was done using genotypic data, trait data, and population structure information using General Linear Models (GLM), and Mixed-Linear Model (MLM) in the software program TASSEL (https://tassel.bitbucket.io/)^89^. Kinship matrix was also used in addition to genotypic data, trait data, and population structure information. The kinship matrix was calculated from genotypic data using the software program TASSEL.

### Analysis of ITS1–ITS4 Sanger sequencing data

The Sanger sequences of ITS1 and ITS4 were manually checked for sequencing errors. The sequences were aligned using the online ClustalW tool (http://www.genome.jp/tools-bin/clustalw) and phylogenetic reconstructions were performed using the function ‘build’ in ETE3 v.3.1.1^90^, as implemented on the GenomeNet (http://www.genome.jp/tools/ete/). The tree was constructed using the function ‘fasttree’ with slow NNI and MLACC = 3 (to make the maximum-likelihood NNIs more exhaustive)^91^. The values at nodes are SH-like local support. To compare these tree results for validity and reliability, MEGA 6 (Build#: 6140226; https://www.megasoftware.net/)^92^ was also used for tree construction. The evolutionary history was inferred using the UPGMA method^93^ and Neighbor-Joining method^94^. The tree is drawn to scale, with branch lengths in the same units as those of the evolutionary distances used to infer the phylogenetic tree. The evolutionary distances were computed using the Maximum Composite Likelihood method^95^ and are in the units of the number of base substitutions per site. The percentage of replicate trees in which the associated taxa clustered together in the bootstrap test (1000 replicates) are shown next to the branches^96^. Codon positions included were 1st + 2nd + 3rd + Noncoding. All positions containing gaps and missing data were eliminated. There were a total of 528 and 537 positions for ITS1 and ITS4 respectively in the final dataset. Also, the aligned sequences of all 37 accessions were analyzed in BioEdit v.7.2.6 (http://en.bio-soft.net/format/BioEdit.html)^97,98^ for SNP identification.

## Supplementary Information

Supplementary Figures.

## Data Availability

Voucher specimen number 3750-KASH of *Sambucus wightiana* was deposited in the KASH herbarium of the University of Kashmir, which was identified by the curator, Mr. Akhtar H. Malik. The study complies with the national and international guidelines. Moreover, appropriate permission was obtained for the collection of plant material.
