# Supplementary material for: Genetic diversity may help evolutionary rescue in a clonal endemic plant species of Western Himalaya
Source: Sci Rep. 2021 Oct 1;11:19595. doi: 10.1038/s41598-021-98648-8 (PMC8486807; doi:10.1038/s41598-021-98648-8)
Supplement: Supplementary file 1 — Supplementary Figures. [file 41598_2021_98648_MOESM1_ESM.docx]

**Title:**

**Genetic diversity may help evolutionary rescue in a clonal endemic plant species of Western Himalaya**

**Authors:**

Irshad Ahmad Sofi^1^, Irfan Rashid^1*^, Javaid Yousuf Lone^1^, Sandhya Tyagi^2^, Zafar A Reshi^1,^ Reyazul Rouf Mir^3*^

^1^ Department of Botany, University of Kashmir, Srinagar – 190006, Jammu and Kashmir, India

^2^ Department of Plant Physiology, Indian Agricultural Research Institute, New Delhi – 110012, Delhi, India

^3^ Division of Genetics & Plant Breeding, Faculty of Agriculture, SKUAST-Kashmir, Wadura Campus, Sopore – 193201, Jammu and Kashmir, India


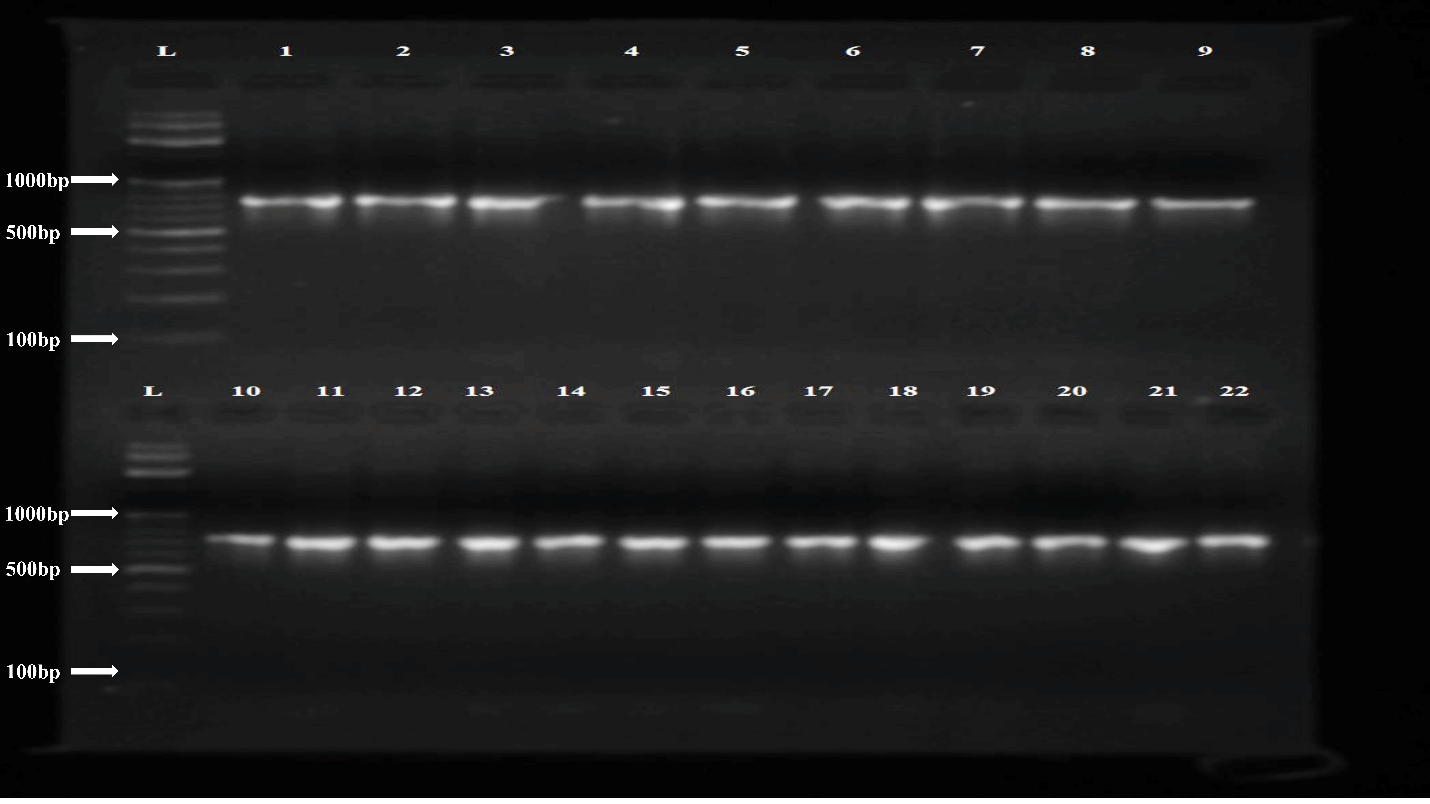


Supplementary Figure S1a: Agarose gel of ITS1-ITS4 amplifications. L = Ladder (100bp DNA Ladder), 1-22 = Samples.


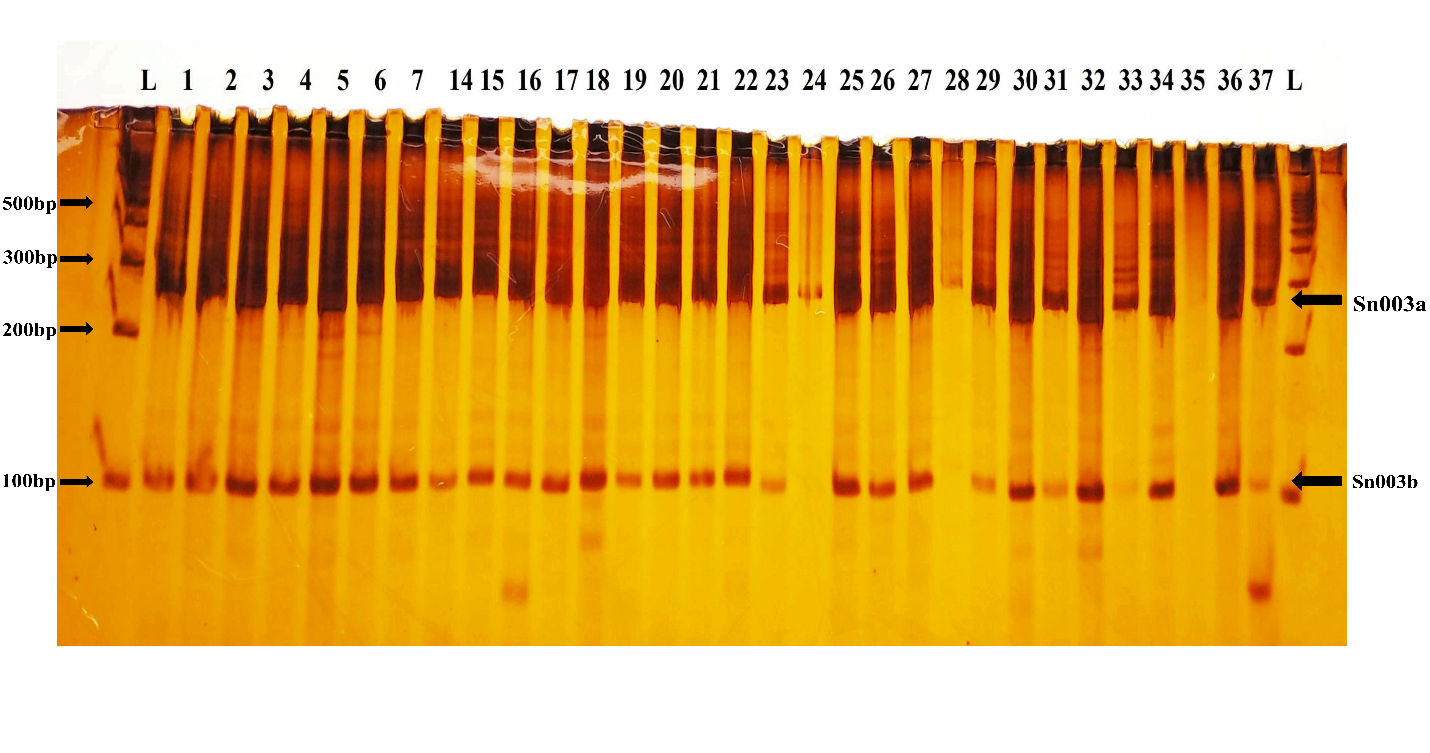


Supplementary Figure S1b: PAGE gel for primer EMSn003. L = Ladder (100bp DNA Ladder), 1-37 = samples. Arrows (right) indicate the 2 loci Sn003a and Sn003b.

Supplementary Figure S2: UPGMA dendrogram showing clustering pattern from ITS1 sequences of *S. wightiana* Samples. The bootstrap values are given on the nodes (MEGA 6, Build#: 6140226; https://www.megasoftware.net/).

Supplementary Figure S3: UPGMA dendrogram showing clustering pattern from ITS 4 sequences of *S. wightiana* samples. The bootstrap values are given on the nodes (MEGA 6, Build#: 6140226; ttps://www.megasoftware.net/).

Supplementary Figure S4: UPGMA dendrogram showing clustering pattern from ITS 1 sequences of *S. wightiana* samples and 17 other species of genus *Sambucus*. The bootstrap values are given on the nodes (MEGA 6, Build#: 6140226; ttps://www.megasoftware.net/).
